# Mechanistic studies of the biogenesis and folding of outer membrane proteins *in vitro* and *in vivo*: What have we learned to date?

**DOI:** 10.1016/j.abb.2014.02.011

**Published:** 2014-12-15

**Authors:** Lindsay M. McMorran, David J. Brockwell, Sheena E. Radford

**Affiliations:** Astbury Centre for Structural Molecular Biology, University of Leeds, Leeds LS2 9JT, UK; School of Molecular and Cellular Biology, University of Leeds, Leeds LS2 9JT, UK

**Keywords:** Protein folding, Outer membrane protein, Periplasmic chaperone, BAM complex, *Φ*-Value analysis, Protein stability

## Abstract

Research into the mechanisms by which proteins fold into their native structures has been on-going since the work of Anfinsen in the 1960s. Since that time, the folding mechanisms of small, water-soluble proteins have been well characterised. By contrast, progress in understanding the biogenesis and folding mechanisms of integral membrane proteins has lagged significantly because of the need to create a membrane mimetic environment for folding studies *in vitro* and the difficulties in finding suitable conditions in which reversible folding can be achieved. Improved knowledge of the factors that promote membrane protein folding and disfavour aggregation now allows studies of folding into lipid bilayers *in vitro* to be performed. Consequently, mechanistic details and structural information about membrane protein folding are now emerging at an ever increasing pace. Using the panoply of methods developed for studies of the folding of water-soluble proteins. This review summarises current knowledge of the mechanisms of outer membrane protein biogenesis and folding into lipid bilayers *in vivo* and *in vitro* and discusses the experimental techniques utilised to gain this information. The emerging knowledge is beginning to allow comparisons to be made between the folding of membrane proteins with current understanding of the mechanisms of folding of water-soluble proteins.

## Highlights

• Summary of key concepts in protein folding from the study of water-soluble proteins.
• Discussion of the complexity of studying folding of outer membrane proteins (OMPs).
• Role of periplasmic chaperones and the BAM complex in the folding of OMPs *in vivo.*
• Examples of the application of biophysical methods to study OMP folding *in vitro.*
• Comparisons of the folding of water-soluble proteins and OMPs.

## Graphical abstract

**Figure:**
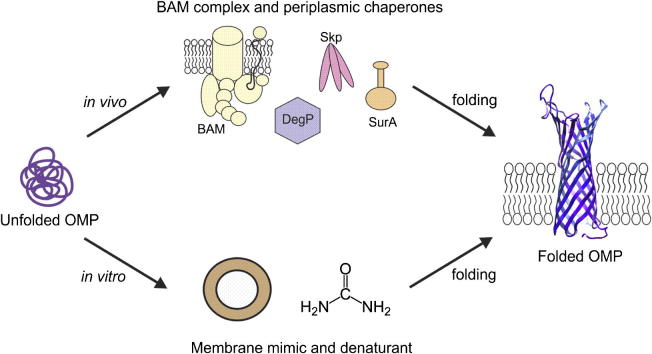

## Principles of protein folding

### How water-soluble proteins fold

The biological activity of many proteins is reliant on their ability to adopt a specific, three-dimensional structure. Failure to achieve this structure can have serious consequences, as evidenced by the prevalence of diseases caused by protein misfolding and aggregation [1], [2]. The information required for a polypeptide chain to attain its native structure was shown to be contained within its primary sequence by Anfinsen’s experiments on ribonuclease A [3], [4]. This small, globular protein was completely unfolded in chemical denaturants and shown to regain its native conformation spontaneously upon dialysis [3]. This seemingly simple conclusion has led to decades of scientific research to determine how the amino acid sequence of a protein confers its native structure.

If folding were to occur by random sampling of all possible conformations of the polypeptide chain, finding the native state would take an astronomically long time. Proteins, however, fold on biologically relevant timescales [5]. Levinthal suggested that this apparent paradox could be resolved if proteins fold *via* defined pathways [6]. Following this conclusion, several mechanisms were proposed to describe the pathways traversed by a protein *en route* to the native state. Analysis of the refolding kinetics of ribonuclease A revealed two distinct phases [7] and led to the suggestion of a “framework” mechanism of folding whereby secondary structural elements of proteins are formed prior to their docking to form the tertiary structure [8], [9], [10]. Further investigation of the refolding of ribonuclease A revealed that one of the phases observed was not, in fact, due to the presence of an observable folding intermediate, but arose as a consequence of proline *cis*–*trans* isomerisation [11]. This realisation, alongside the characterisation of the folding of chymotrypsin inhibitor 2 which revealed a simple two-state folding mechanism [12], [13], [14], saw the framework mechanism become disfavoured due to its implication that folding intermediates should be present [10], [15]. To explain folding in the absence of detectable intermediates, the nucleation-condensation mechanism was proposed by Fersht and co-workers [12]. This mechanism involves the formation of a small nucleus of structure stabilised by weak, possibly long-range, contacts and the subsequent rapid collapse around this folding nucleus to yield the native state [10], [12], [15]. A combination of simulation and experimental data on members of the homeodomain-like super-family of proteins revealed that the seemingly contrasting framework and nucleation-condensation folding models could be considered as two extremes of a single mechanism. In this unified model, the relative stability of the secondary and tertiary structure determines whether these elements are formed in series or in parallel [16] and, thus, two decades of conflicting evidence were resolved.

**Fig. 1:**
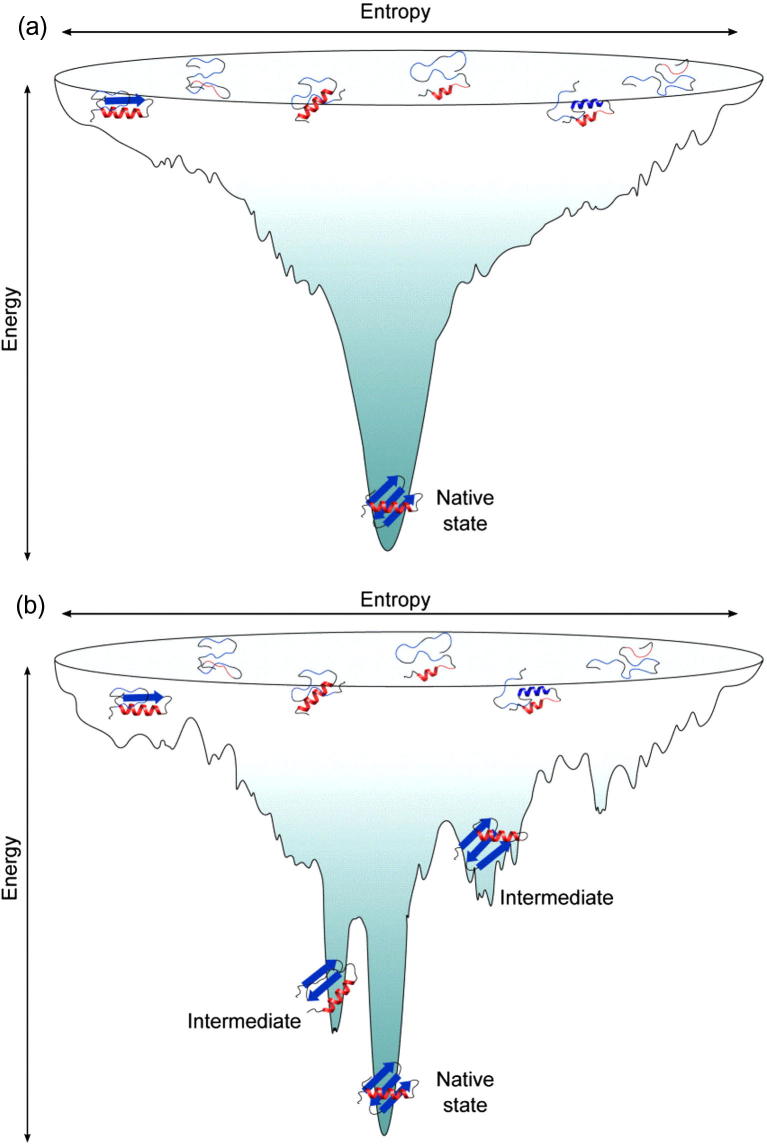
Schematic representation of funnel-shaped folding landscapes. Example of (a) a smooth folding landscape expected for a two-state folding mechanism where only the native and unfolded states are stably populated and (b) a rugged landscape wherein the polypeptide chain populates one or more intermediate structures which represent local energy minima. Reprinted from [31] by permission from Macmillan Publishers Ltd: Nat. Struct. Mol. Biol. © 2009.

More recently it has been realised that proteins fold *via* a collection of parallel pathways which make up a funnel-shaped energy landscape (Fig. 1) [17], [18], [19], [20]. At the top of the funnel, the unfolded state represents a large ensemble of high-entropy conformations of the polypeptide chain. While unstructured, the polypeptide chain may be biased by weak, residual interactions which initiate folding [21]. Indeed, an unfolded variant of the bacterial immunity protein Im7 has been studied recently under non-denaturing conditions, revealing conformational restriction in the regions of the protein sequence which ultimately form the native helices, emphasising the importance of such interactions in the initiation of folding [22]. Similar conclusions have been drawn from other proteins and protein fragments under different denaturing conditions [23], [24], [25], [26], [27], [28]. As folding progresses, the polypeptide chain undergoes many reorganisations aiding the formation of stabilising interactions between side-chains, the protein backbone and the solvent as the protein approaches the native state [17]. The landscape view is an attractive one as it does not place restrictions on whether secondary structure must form before, or at the same time, as the tertiary structure. Additionally, the funnel-shaped landscape predicts the experimentally observed robustness of the folding process to destabilising mutations: if the final fold remains the most stable state relative to the unfolded ensemble, a mutation may block some of the pathways to the native state but alternative folding pathways can be utilised [17].

The landscape theory of protein folding also predicts the existence of “downhill folding”, that is, folding in the absence of significant energy barriers [17]. Even with the most powerful single molecule techniques available it is still difficult to experimentally identify barrier-less folding unequivocally [29]. Exciting developments in simulation methods have recently enabled the first visions of protein folding in all-atom detail for small, single domain proteins [30]. These simulations have suggested that at least 9 of the 12 rapidly-folding proteins studied experience energy barriers that slow folding [30]. For proteins in which only the native and unfolded states are stably populated, known as a two-state folding mechanism, the folding energy landscape is relatively smooth (Fig. 1a) [31]. With the advent of more rapid triggering methods (ultra-rapid mixing, temperature jump, *etc.* [31]) and more sensitive detection methods (e.g. single molecule techniques [32], [33], [34]), such a simple folding scenario is rarely observed, even for small proteins. Instead, the folding landscape is often rugged with local energy minima representing the population of one or more folding intermediates *en route* to the native state (Fig. 1b) [31], [35].

Rugged folding landscapes result as a consequence of the need to form the thousands of weak, stabilising interactions which are present in the native state (reviewed in Ref. [31]). During the structural reorganisations that are required for the protein to traverse the folding landscape, it is not always possible to minimise the energy of all of these interactions simultaneously, leading to “frustration” in the landscape [19], [36]. Theory predicts that a rough landscape will lead to slower folding by limiting the rate at which the protein can find the native state [19]. Recently the effect of solvent viscosity on the folding and unfolding kinetics of homologous domains of α-spectrin was studied, revealing the first explicit experimental evidence that differences in internal friction, and hence frustration, can indeed influence the folding kinetics of structurally homologous proteins [37]. It has been suggested previously that frustration, and hence ruggedness of energy landscapes, arises due to conflicting pressures to evolve protein sequences which can reliably fold to a stable native structure and that are capable of carrying out a defined biological role [19], [36]. While this may be the case for some proteins, the differences in unfolding rates of structurally and functionally similar α-spectrin domains were shown to be a consequence of different degrees of landscape ruggedness, with slower unfolding occurring as a result of increased frustration [37]. This result suggests that frustration has been selected to slow unfolding in this protein domain, which has an unusually long half-life *in vivo* [37]. Folding of the Trp-cage mini protein was also shown to be slowed when its folding intermediate was destabilised by the presence of chemical denaturant or a helix-breaking mutation [38]. This suggests that local energetic minima in the folding landscape can act to separate the conformational search into multiple, smaller problems and hence accelerate the conformational search process (reviewed in Ref. [35]). Whether folding intermediates act to disrupt or promote the folding process for many proteins is still under debate. The utility of intermediate species in providing insights into the structural regulation of biological function as well as their role in the initiation of protein aggregation and recognition by molecular chaperones, makes them important targets of study [39].

This brief overview of the folding of small water-soluble proteins has outlined how modern advances in experimental and computational techniques have provided significant progress towards understanding the folding and assembly mechanisms of this class of proteins. Many fundamental questions remain unanswered about this complex and important biological process, particularly regarding the folding of more complicated systems such as multi-domain proteins, folding in the cellular milieu and membrane protein folding [40], [41], [42], [43], [44]. Current research into understanding the latter problem, focussing on bacterial outer membrane proteins (OMPs), is covered in the following sections.

### The membrane protein folding problem

Understanding the folding, stability and function of membrane proteins is an important area of research as these proteins represent 60% of current drug targets and have vital roles in the cell, including signalling, transport and biogenesis [45], [46]. In contrast with the wealth of information available about the folding of small, water-soluble proteins, the field of membrane protein folding has lagged significantly [47]. For water-soluble proteins, folding is driven by the need to bury hydrophobic side-chains in order to prevent aggregation and to facilitate the formation of stable structures [40]. In addition to the attainment of the native state, the folding of integral membrane proteins is complicated by the need to insert the polypeptide chain into a lipid membrane [47]. Following membrane insertion, most of the surface of an integral membrane protein is in contact with the membrane’s hydrophobic interior. Hydrophilic residues are either restricted to regions which contact the polar head groups of the membrane lipids or are exposed to the aqueous environment on either side of the membrane [47]. Additionally, the membrane environment *in vivo* is highly dynamic and heterogeneous with regions of varying lipid composition [48]. Recreating this environment *in vitro* has proved more difficult than the simple aqueous environment needed to fold water-soluble proteins and this, in part, has limited studies on the folding of integral membrane proteins [47], [48].

## Classes of membrane proteins

**Fig. 2:**
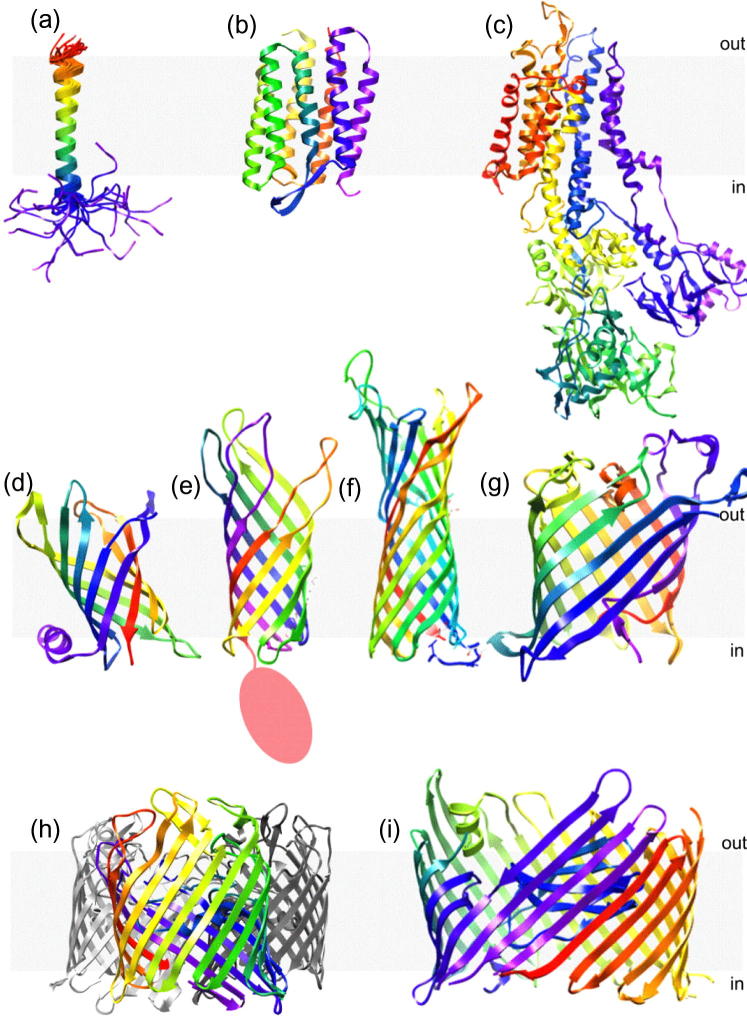
Example structures of integral membrane proteins. Structures of (a) the transmembrane segment of a glycophorin A monomer from human erythrocyte membranes solved by NMR spectroscopy (1AFO [228]); (b) bacteriorhodopsin, a seven-helical bundle from the purple membrane of *Halobacterium salinarum* (1C3 W [229]); (c) calcium ATPase 1 from the sarcoplasmic reticulum membrane of *Oryctolagus cuniculus*, a ten-helical bundle with a large cytoplasmic domain (1IWO [230]); (d) PagP, an 8-stranded palmitoyl transferase enzyme from *E. coli* (1THQ [206]); (e) the 8-stranded transmembrane domain of OmpA, an ion channel from *E. coli* (1BXW [231]), with the C-terminal periplasmic domain (structure currently not determined) represented by a red circle; (f) the 10-stranded OM protease, OmpT, from *E. coli* (1I78 [232]); (g) the 12-stranded, colicin-secreting phospholipase A, OmpLA, from *E. coli* (1QD5 [233]); (h) the OmpF porin, a trimer comprised of three 16-stranded β-barrels, from *E. coli* (2ZFG [234]) and (i) the 24-stranded translocation domain of PapC from *E. coli* (3FIP [235]). Unless otherwise specified, all structures were solved using X-ray crystallography. Proteins are coloured rainbow: violet (N-terminus) to red (C-terminus). In (h), a single OmpF monomer is coloured, while the remaining monomers are shown in greyscale. The approximate position of the membrane is indicated in all images with grey shading. All images were generated from the PDB files using the accession numbers given in brackets using UCSF Chimera molecular visualisation application [236].

The proteins present in biological membranes can be categorised into two families: the lipid-anchored proteins, which have a covalently-bound fatty-acid moiety through which a water-soluble protein is attached to a membrane, and the integral membrane proteins, which contain membrane-spanning regions. Only the folding mechanism of the latter will be described here. In contrast with lipid-associated proteins, the integral membrane proteins are constrained by the need to compensate for the energetic cost of burying peptide bonds in the lipid bilayer [49], estimated to be 1.2 kcal/mol per peptide bond [50]. As a consequence, it was predicted that membrane spanning regions would form regular secondary structural elements in order to maximise the hydrogen bonding potential of the peptide backbone [49]. Formation of secondary structure reduces the energetic cost of incorporation of peptide bonds into a bilayer by ≈0.4 kcal/mol per peptide bond for α-helical structure and ≈0.5 kcal/mol per peptide bond for β-sheet structure [50]. The first α-helical membrane protein structure was solved in 1975 by Henderson and Unwin using electron microscopy to generate a three-dimensional image of the purple membrane of *Halobacterium salinarum* [51]. The resulting 7 Å resolution image revealed the structure of bacteriorhodopsin (bR)^1^ to be a seven helical, transmembrane bundle [51]. The structural information available about bR has since been increased by structures at higher resolution obtained using electron microscopy (3 Å, [52]) and X-ray diffraction (1.43 Å, [53]). Since the structural elucidation of bR, a wide variety of helical transmembrane structures have been solved and deposited in the Protein Data Bank (PDB) [54]. These show a diverse range of size and function across the kingdoms of life. Some examples are depicted in Fig. 2a–c.

In contrast with the ubiquitous distribution of α-helical transmembrane proteins, membrane proteins containing β-sheet secondary structure are found only in the outer membranes of Gram-negative bacteria, mitochondria and chloroplasts [55], [56]. In order to satisfy all of the possible hydrogen bonds in the peptide backbone, each β-strand associates laterally with its neighbours resulting in an overall cylindrical topology, known as a β-barrel [55]. With predominantly non-polar side chains exposed to the hydrophobic membrane interior and each of the backbone hydrogen bonds satisfied, the resulting β-barrel structures have high thermodynamic stability [50], [57]. The size of the β-barrel is highly variable, with known structures containing between 8 and 24 β-strands, and the proteins may contain periplasmic or extracellular domains [55], [56]. The regions between β-strands often alternate between tight turns on the periplasmic side of the membrane and longer, more flexible loops on the outer surface, which are exposed to the external environment (Fig. 2d–i) [55]. One almost entirely conserved structural feature across this family of protein structures is an even number of β-strands, with only one known exception to this rule discovered thus far: the 19-stranded human mitochondrial voltage-dependent anion-selective channel (hVDAC) [56], [58].

As more and more research is focused on the determination of membrane protein structure, a total of over 430 unique structures have now been reported in the membrane protein structure (mpstruc) database with both α-helical and β-barrel proteins represented [59]. Structure determination has been most successful using X-ray diffraction, although 105 of the reported structures were solved using nuclear magnetic resonance (NMR) spectroscopy and a further 14 structures have been solved using electron diffraction, demonstrating the utility of multiple techniques in the membrane protein structure determination tool box [60], [61]. Almost 30 years after the first structure was reported, integral membrane proteins still account for only 1% of the protein structures in the PDB [62]. Significant progress is being made in this area, however, with 64 structures determined in 2012 and 50 in 2013 to date [59].

## Biological membranes

Lipids in cells have three main functions: energy storage, signal transduction and forming the matrix of biological membranes – the approximately 30 Å thick layer which encloses the cell and organelles within eukaryotic cells [63]. Cellular membranes are composed of polar lipids, which self-associate into bilayers to shield the hydrophobic regions from the aqueous environment in a process driven entropically by water molecules [63]. The structure and composition of lipid bilayers varies greatly even within an organism, allowing the properties of different membranes to be tailored to a specific function. Modulation of lipid composition to adapt to different functional requirements implies the evolutionary advantage of an extensive and complex lipid repertoire [63]. In addition to varying lipid composition, membranes can have either a symmetrical or asymmetrical distribution of lipids between the two leaflets of the bilayer. Within a leaflet, favourable interactions between some of the lipid components can generate domains of specific lipid composition, known as lipid rafts, which are thought to be involved in localising membrane proteins [63]. The variable properties of biological membranes demonstrate the highly dynamic, heterogeneous and complex nature of the lipid environment in which integral membrane proteins must insert, fold and function.

## The cell envelope of Gram-negative bacteria

**Fig. 3:**
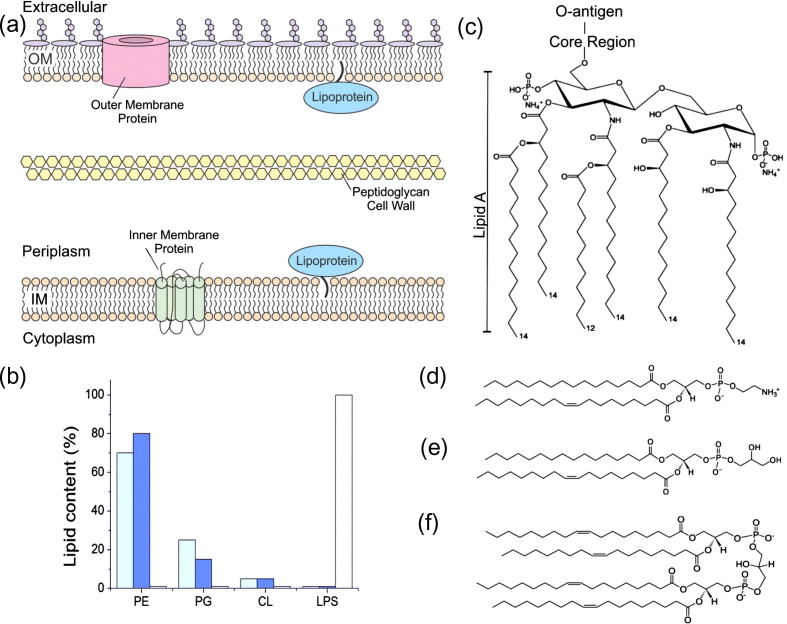
The cell envelope of Gram-negative bacteria. (a) The cytoplasm of *E. coli* is surrounded by the inner membrane (IM), the periplasm and the outer membrane (OM). The IM is a symmetric phospholipid (shown in orange) bilayer containing integral α-helical membrane proteins. The OM is an asymmetric bilayer of phospholipid and lipopolysaccharide (LPS, shown in purple) and contains β-barrel integral membrane proteins. The periplasm is the aqueous compartment between the two membranes in which the peptidoglycan cell wall is found. Both membranes have associated lipoproteins on their periplasmic faces. (b) The lipid composition of the IM (light blue), inner leaflet of the OM (dark blue) and outer leaflet of the OM (white) in *E. coli* (percentages based on those reported in [64], [65], [66]). Structures of (c) LPS, (d) phosphatidylethanolamine, (e) phosphatidylglycerol and (f) cardiolipin are shown.

A hallmark of Gram-negative bacteria, such as *Escherichia coli*, is the cell envelope, which is composed of two lipid membranes enclosing an aqueous compartment called the periplasm (Fig. 3a) [64], [65]. The inner membrane (IM) is a symmetric phospholipid bilayer composed of approximately 70% phosphatidylethanolamine (PE, Fig. 3d), 25% phosphatidylglycerol (PG, Fig. 3e) and 5% or less cardiolipin (Fig. 3f) and forms the barrier between the cytoplasm and the periplasm (Fig. 3a) [64], [65], [66]. There are two types of protein associated with the IM; lipoproteins which undergo lipid modifications of an N-terminal cysteine residue to anchor them to the periplasmic face of the IM and the α-helical integral membrane proteins (reviewed in Ref. [65]). IM proteins are responsible for many cellular processes, including lipid synthesis and small molecule transport. This class of proteins is discussed at length elsewhere in this issue.

The periplasm is the compartment between the IM and the outer membrane (OM), which comprises around 10% of the total cell volume and contains soluble proteins, as well as a peptidoglycan cell wall. The peptidoglycan layer plays important roles in maintaining the shape of the cell and preventing lysis, while periplasmic proteins are involved in maintaining the integrity of the cell envelope. The processes which take place in the periplasm are independent of nucleotide hydrolysis, since no ATP is present in this compartment [65], [67]. When energy is required, the cell relies on complex coupling mechanisms which often use the proton-motive force across the inner membrane as the primary energy source [65].

The inner leaflet of the asymmetric OM is comprised of phospholipids and is similar in composition to the IM, although the PE content is enriched compared with the IM (Fig. 3b) [65], [66]. The outer leaflet of the OM consists of lipopolysaccharide (LPS), a glycolipid typically consisting of lipid A, a core oligosaccharide and an O-antigen (Fig. 3c) [64], [68]. The structure of the O-antigen is highly variable, even within a species, with approximately 170 variants being recorded in *E. coli* [69]. The high number of fatty acid chains on LPS compared with phospholipids, and the fact that these chains are saturated, facilitates tight packing of the LPS molecules in the OM [68]. This confers low fluidity to the membrane and it is this property that is responsible for the low permeability of the OM [68]. As a consequence, rapid diffusion of small, hydrophobic molecules across the OM is prevented and Gram-negative bacteria thus tend to be less susceptible to hydrophobic antibiotics than their Gram-positive counterparts [68]. As with the IM, integral membrane proteins and lipoproteins are associated with the OM, but in this membrane the integral outer membrane proteins (OMPs) usually have β-barrel structures [55], [65]. The barrel is formed by membrane-spanning β-strands which are held together by a lateral hydrogen bond network [55]. One reported exception, the polysaccharide translocon Wza of *E. coli*, has a barrel structure composed of laterally associated α-helices [70]. Several examples of OMP structures are shown in Fig. 2d–i.

## OMP biogenesis *in vivo*

### Periplasmic chaperones assisting OMP biogenesis

**Fig. 4:**
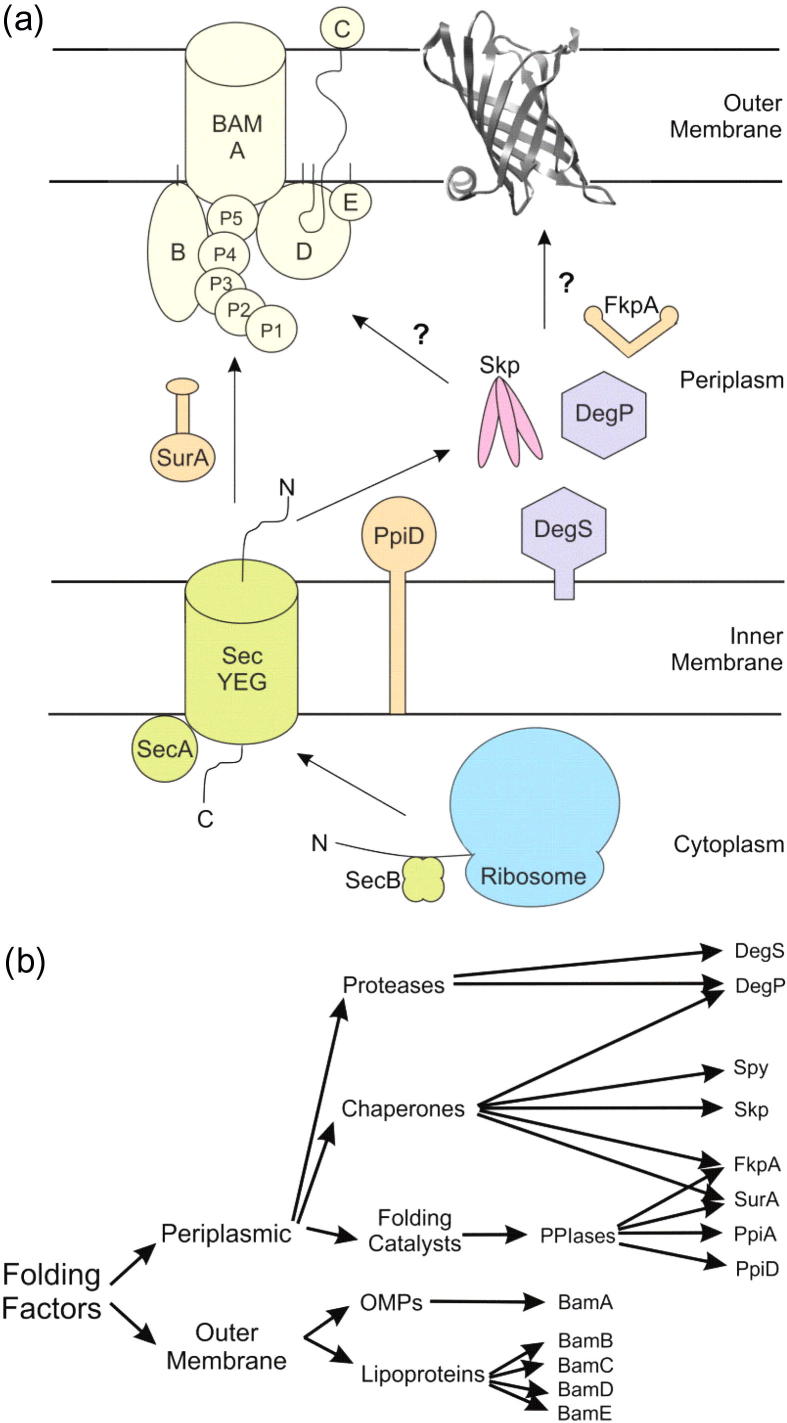
Schematic of the current model of biogenesis and chaperoning of OMPs in *E. coli.* (a) OMPs are synthesised on the ribosome before post-translational translocation across the inner membrane by the SecYEG translocon. Unfolded OMPs are then chaperoned across the periplasm to the β-barrel assembly machinery (BAM) complex, which aids folding and insertion into the OM. BAM complex proteins are labelled A–E, and the periplasmic polypeptide transport-associated (POTRA) domains of BamA are labelled P1-5. Horizontal black lines indicate the approximate position of the inner and outer membranes*.* (b) Flow diagram of the periplasmic and outer membrane-anchored proteins which may be implicated in OMP biogenesis. (a) was adapted from [92] with permission from Elsevier, © 2013, while (b) was reproduced from [73] with permission from John Wiley and Sons, © 2005.

Following their synthesis in the cytosol, OMPs are targeted to the SecYEG translocon by the SecB chaperone, whereupon they are translocated across the IM through SecYEG in an unfolded state [71], [72]. The unfolded OMPs must be protected from aggregation and must be able to traverse the periplasm, including the peptidoglycan layer, and then correctly fold and insert into the OM [73]. These requirements suggest that transport across the periplasm and membrane insertion may be facilitated processes and, indeed, a number of periplasmic and OM-associated proteins have been implicated in the OMP assembly pathway [73]. These proteins can be roughly grouped into three categories: proteases; chaperones which stabilise unfolded and non-native conformations of their client proteins; and folding catalysts, which catalyse rate-limiting steps in folding (Fig. 4a and b) [73].

Given the range of essential functions carried out by OMPs [56], it is unsurprising that the presence of unfolded OMPs in the periplasm is a strong inducer of the envelope stress response [73]. Exposed C-terminal residues of misfolded OMPs are recognised by the PDZ domain of the inner membrane-associated protease, DegS (Fig. 4a), activating the protease domain [74]. Activated DegS triggers a proteolytic cascade which leads to induction of the σ^E^ heat-shock response [73], [74]. Using the assumption that depletion of periplasmic chaperones will cause an increase in misfolded OMPs, identification of many of the known periplasmic folding factors arose by genetic studies of bacterial strains showing high σ^E^ activity [67]. Some of the key periplasmic assistants of OMP folding, including SurA, Skp, DegP and FkpA (Fig. 4), are described in the following sections.

#### SurA

SurA was first identified in 1990 when it was shown to be required for the survival of *E. coli* in the stationary phase [75]. Initial characterisation described SurA as a parvulin-like peptidyl-prolyl isomerase (PPIase) involved in the proper assembly of major OMPs [67], [76], [77]. Trypsin digestion of OMPs in *surA* deletion mutants showed that not all OMPs have increased rates of proteolysis, leading to the conclusion that SurA is not an essential folding factor [76]. The amounts of FadL, LamB, OmpA, OmpC, OmpF, OmpX and LptD, however, were all found to be reduced in *surA* deletion strains [78]. Similar results were obtained using a proteomic analysis, which also revealed an upregulation of proteins under the control of the σ^E^ regulon in *surA* deletion strains [79].

**Fig. 5:**
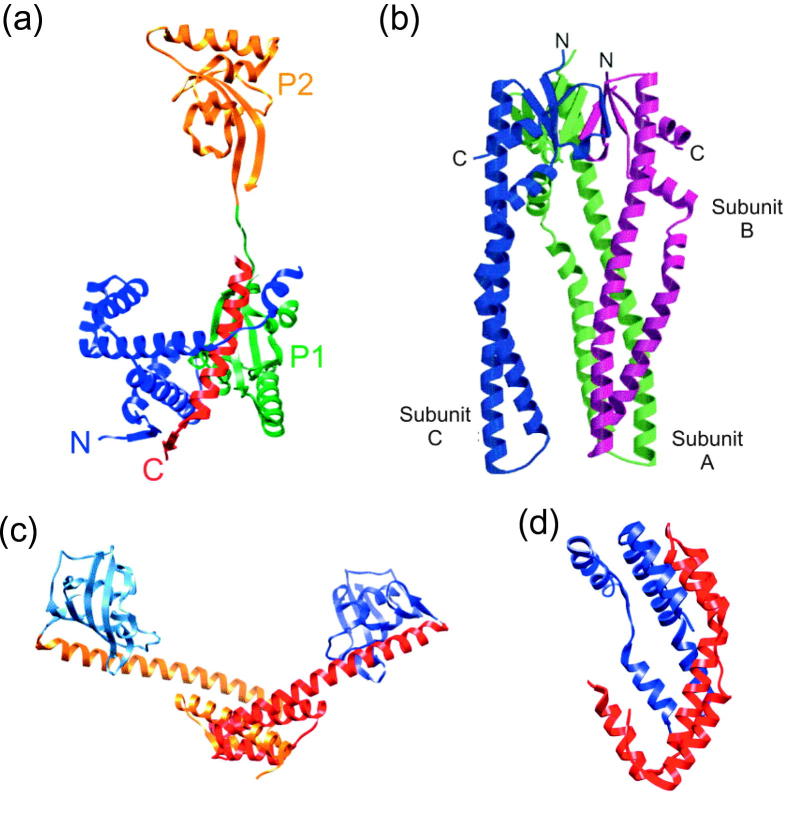
Crystallographic structures of selected periplasmic chaperones. (a) Ribbon diagram of SurA coloured as follows N-terminal domain (blue), PPIase domain P1 (green), PPIase domain P2 (orange) and C-terminal domain (red) (1M5Y [80]). (b) Ribbon diagram of Skp trimer with the subunits A, B and C coloured in green, magenta and blue, respectively, (1U2M [104]). The tips of the α-helices in subunits A and B have been modelled. (c) Ribbon diagram of the FkpA dimer showing the N-terminal chaperone domains (red and orange) through which dimerisation occurs and the C-terminal PPIase domains (blue) (1Q6H [109]). (d) Ribbon diagram of the Spy dimer with the monomers coloured individually in red and blue (3O39 [114]). (a), (c) and (d) were generated from PDB files using the accession numbers given in brackets using UCSF Chimera molecular visualisation application [236]. (b) was reproduced from [104] with permission from Elsevier, © 2004.

Crystallisation of SurA revealed a four-domain protein with two PPIase domains (P1 and P2) sandwiched between the N- and C-terminal domains (Fig. 5a) [80]. PPIase domain P1 is packed against the core structure of the N- and C-terminal domains and does not show significant activity, while the more active P2 domain extends away from the core structure [77], [80], [81]. The PPIase activity of P2 has been shown to be increased in the presence of the adjacent chaperone domain, presumably as this domain facilitates substrate binding close to the active site of P2 [82]. Deletion of both PPIase domains, however, did not cause a significant loss of SurA function *in vivo* and the isolated PPIase domains failed to complement activity in *surA* deletion mutants [81]. This led to the conclusion that SurA functions mainly as a chaperone [81]. Interestingly, mutations which would be expected to cause a loss of PPIase function in the P1 domain, if this domain were active, destabilised SurA *in vitro* but increased chaperone activity *in vivo* [83]. This result suggests a regulatory function of the P1 domain, explaining its lack of significant PPIase activity [83].

Binding studies using peptide sequences have been carried out on SurA *in vitro*, revealing a preference for aromatic-rich sequences with an Ar-X-Ar motif [84], [85]. Peptide sequences containing this motif bind to SurA with dissociation constants in the micromolar range [84], suggesting that SurA binds to OMPs by recognition of this motif, which is frequently found in OMP C-terminal regions [78], [86]. The C-terminal Y-X-F motif is responsible for activating DegS and triggering the σ^E^ stress response [73], [78] and, interestingly, it has been reported that SurA does not show significant binding affinity to this motif [87]. In contrast with the peptide binding data, only a few studies have reported the binding of full length OMPs to SurA [88], [89], [90], [91] and one study failed to detect stable SurA:OMP complexes [92]. The available data, however, suggest that unfolded OMPs are bound by SurA in preference to unfolded soluble proteins, while folded proteins do not bind [89]. While the polypeptide binding site of SurA has not been identified conclusively, an extended crevice, located in the core region, was observed in the crystal structure of SurA. This is thought to be the site through which SurA:client interaction occurs [80].

Sklar and co-workers demonstrated that depletion of SurA causes a loss of OM density, suggestive of OMP assembly defects, that was not observed when the other periplasmic folding factors, Skp and DegP, were depleted [93]. Additionally, SurA has been shown to be localised at the OM [84] and is the only periplasmic folding factor to have been successfully cross-linked to BamA of the β-barrel assembly machinery (BAM) complex *in vivo* [93]. It is not yet known, however, if SurA binds directly to BamA, or if this interaction is mediated through SurA-bound substrates [86]. Together, these observations have led to the hypothesis that SurA is the main chaperone for OMP transport *in vivo* [93], [94], although there is clearly much to learn about the function of this protein at both cellular and molecular levels.

#### Skp

Although Skp had previously been suggested to be a DNA-binding protein, an OMP and an LPS binding protein, it is now known that Skp is a periplasmic protein, as evidenced by the N-terminal signal sequence which targets Skp for translocation across the inner membrane [73]. Depletion of Skp *in vivo* led to a moderate reduction of OmpC, OmpF, OmpA and LamB in the OM fraction [95]. These cells, however, were still viable demonstrating that Skp is not essential. Further genetic studies revealed that both the *skp surA* deletion mutant and the *degP surA* deletion mutant result in a synthetic lethal phenotype, leading to the prevailing hypothesis that the periplasmic chaperones Skp and DegP act on redundant pathways seperate to SurA (Fig. 4a) [94]. Proteomic analysis of a *skp* deletion mutant suggested that none of the OMPs identified were affected significantly; however, depletion of SurA in the *skp* null strain reduced the levels of almost all OMPs, consistent with the hypothesis of parallel chaperone pathways [96]. While this hypothesis explains many of the observations above, the accumulation of protein aggregates in the periplasm of *skp degP* deletion mutants suggests that Skp may have a role in maintaining the solubility of at least some OMPs prior to folding [97]. Additionally, *skp fkpA* deletion mutants showed defects in the folding of LptD, an essential OMP involved in LPS synthesis [98]. Over-expression of SurA could not ameliorate these effects [98]. Together, these results suggest that the chaperone pathways in the periplasm may be inter-dependent.

The chaperone activity of Skp in OMP folding was confirmed recently by the finding that Skp binds with high affinity (*K*_d_∼ nM) to unfolded OMPs [95], [99], [100]. Furthermore, Skp binding to OmpA was shown to occur *via* the transmembrane domain of OmpA [95]. NMR spectroscopy confirmed that the transmembrane domain of OmpA is Skp bound, while the periplasmic domain is free to fold independently in solution [100]. Interaction of unfolded OMPs with Skp is thought to occur early after translocation across the IM, as evidenced by the ability to cross-link Skp to the OMP PhoE at the periplasmic side of the IM in spheroplasts [101]. Skp has also been shown to be required for the release of newly-translocated OmpA from the periplasmic side of the IM in spheroplasts [97], suggesting that the N-terminal residues of the client protein may interact with Skp as they emerge from the translocon. This hypothesis was supported by Förster resonance energy transfer (FRET) experiments which showed that the N-terminal residues of OmpC enter the Skp cavity first [102]. Molecular dynamics (MD) simulations yielded similar results [102].

Skp is a trimeric protein in solution with a structure that has been described as “jellyfish-like” (Fig. 5b) [103], [104]. A β-barrel domain forms the body of the jellyfish, with long α-helical “tentacles” protruding from the β-barrel [103], [104]. Skp is a basic protein, with a theoretical isoelectric point (pI) around 9.5 [73]. The charge distribution of Skp is notable, with many basic residues clustered around the tips of the α-helical tentacles resulting in a large macrodipole moment [103], [104]. This unusual distribution of charges suggests a role of Skp in substrate delivery to negatively charged membranes. Additionally, a putative LPS-binding site was identified on the outer edge of the Skp α-helices, similar to an LPS-binding motif found on the OMP FhuA [104]. This finding led to the suggestion that Skp may act as a stress chaperone, acting to rescue OMPs which veer off the SurA chaperone pathway [105].

The α-helical domains of Skp define a central cavity enriched with hydrophobic residues, which has been shown to be involved in sequestering unfolded substrates to prevent their misfolding and aggregation [88], [90], [100], [102], [103], [104]. In contrast with SurA, no common binding motif has been identified for the interaction of Skp with its substrates [73], [90] and both electrostatic and hydrophobic interactions have been implicated as being important for binding [99], [102], [106]. In the absence of a specific binding motif, the high affinity interaction between Skp and its substrates has been shown to arise from the formation of a large number of simultaneous weak interactions that exist between the chaperone and its substrate [90]. Despite a low sequence identity, Skp shares a striking structural resemblance to prefoldin [104], an ATP-independent, cytosolic chaperone in eukaryotes and archaea, which protects partially folded proteins from aggregation and passes clients to other chaperones to promote folding [103], [104]. The transient nature of the multitude of weak, local interactions between Skp and its substrates has also been hypothesised to facilitate handover of substrates to other chaperones [90], which could point to functional, as well as structural similarities, between these ATP-independent chaperones. Conformational analysis of Skp-bound OmpX revealed a highly flexible, but compact, ensemble which lacks secondary structure [90], in contrast with the molten globule-like conformations of substrates bound by ATP-dependent chaperones such as GroEL [107]. This difference in substrate binding provides further evidence that ATP-independent chaperones, such as Skp, rely on a thermodynamic gradient for substrate release and subsequent folding [90], [108]. In this model, the large free energy of folding of OMPs provides a “thermodynamic sink” that drives the release of these client proteins from Skp and enables their subsequent folding in the OM [108]. The ability of Skp to prevent aggregation of water-soluble proteins has also been shown using clients including lysozyme [104] and single chain antibodies [106], revealing that Skp is able to chaperone soluble proteins as well as OMPs. Skp may, therefore, act as a universal chaperone assisting the folding of both soluble proteins and OMPs in the periplasm.

#### Other periplasmic folding factors

While SurA and Skp are currently the most studied of the periplasmic folding factors, other proteins have been shown to participate in OMP biogenesis. Three other periplasmic PPIase enzymes are currently known: PpiA, PpiD and FkpA [64]. While PpiA has been shown to have the highest activity of these proteins *in vitro*, its deletion had no detectable effect on the assembly of OMPs *in vivo* and, as yet, no evidence has directly linked PpiA to OMP biogenesis [64], [73]. In contrast, deletion of the inner membrane-anchored PpiD (Fig. 5a) was reported to cause a reduction in the levels of OmpC, OmpF, OmpA and LamB in the OM of the mutant cells and a *ppiD surA* deletion mutant was reported to be lethal, implicating PpiD in OMP folding [64], [73]. Later studies did not replicate these findings, however, and the role of PpiD in OMP assembly remains an open question [64].

FkpA, like SurA, is an example of a dual PPIase-chaperone folding factor and *fkpA* null mutants have been shown to be viable, but to display increased OM permeability and an up-regulation of periplasmic proteases [67]. Structural studies revealed that FkpA has a C-terminal PPIase domain, appended to an N-terminal chaperone domain that mediates dimerisation to form a v-shaped cleft (Fig. 5c) [109]. It has been hypothesised that substrate binding occurs in this cleft [109], but no data are currently available to support this. Until recently, chaperone activity of FkpA had been reported only for soluble protein substrates and the involvement of FkpA in OMP biogenesis was not well supported [64], [109]. Creation of a *skp fkpA* deletion mutant by Schwalm and co-workers, however, showed that folding of LptD is compromised in this strain, providing the first evidence that FkpA may indeed chaperone OMPs in the periplasm [98].

Another example of a dual-function chaperone in the periplasm is the protease-chaperone DegP which, alongside DegS, belongs to the HtrA family of proteases [73]. Temperature changes were initially thought to cause the switch between the two activities of DegP, with the chaperone activity dominating at 28 °C and the protease activity becoming dominant at 42 °C [73]. Structure–function analysis of DegP concluded that the resting state of this protein is a hexamer, in which the interactions between subunits block the protease sites and the central cavity is large enough to accommodate unfolded substrates [110]. Similarly to DegS, DegP recognises the C-terminal residues of misfolded OMPs *via* its PDZ domains and this was thought to cause structural reorganisation to larger proteolytically-active oligomers comprised of 12 or 24 DegP monomers [78], [111], [112]. Cyro-electron microscopy of DegP in the presence of OmpC revealed a tetrahedral arrangement of DegP trimers with the inner cavity filled by a cylindrical area of electron density into which the structure of folded OmpC could be modelled [111]. This led to the conclusion that the fate of DegP-bound substrates lies in their ability to adopt their native structure within the cavity, as only unfolded substrates can be degraded [111]. Recent data using cage-deficient mutants of DegP, which can only associate into trimers, revealed that these variants are able to bind and degrade substrates without the need to form higher order oligomers, suggesting that cage formation in response to substrate binding may be linked solely to the chaperone function of this protein [113].

The most recently discovered periplasmic chaperone, Spy, was identified by its over-expression in bacterial strains expressing unstable periplasmic proteins [114]. *In vitro* characterisation of Spy revealed it to be an effective chaperone, suppressing aggregation and protecting substrates from inactivation by tannins [114]. Spy has a novel α-helical cradle structure, which is unlike that of any known chaperone (Fig. 5d) [114]. It is not known, currently, whether Spy is involved in OMP biogenesis, but the discovery of a previously unknown chaperone only two years ago highlights that there is much still to be learned about the chaperone network in the *E. coli* periplasm. In addition to the complexity evident in a single species, studies on the periplasmic chaperones of *Neisseria* *meningitides* show that SurA deletion causes no detectable defects in OMP assembly, while deletion of Skp caused lower levels of some porins, but not all OMPs [115]. Complementation of the *skp* null mutant was not possible using *E. coli* Skp, suggesting that periplasmic chaperones may act in a species-specific manner, making the derivation of generic principles of periplasmic chaperoning difficult to achieve [115].

### Insertion into the outer membrane

**Fig. 6:**
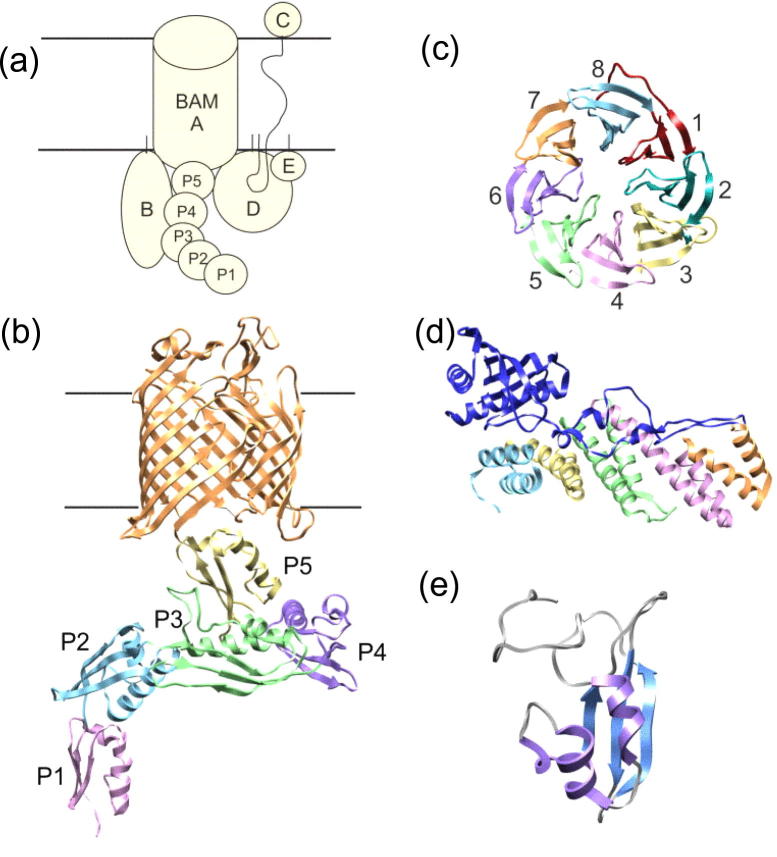
Structure of the BAM complex. (a) Schematic of the *E. coli* BAM complex with BAM proteins labelled A–E and POTRA domains labelled P1–5 (reproduced from [92] with permission from Elsevier, © 2013). (b) Crystal structure of *N. gonorrhoeae* BamA (4K3B [120]). The β-barrel domain is shown in orange. POTRA domains are labelled as in (a) and are shown in pink, blue, green, purple and yellow. (c) Crystal structure of *E. coli* BamB (3P1L [130]). The blades of the β-propeller structure are coloured individually and labelled 1–8. (d) Crystal structure of the N-terminal domain of *E. coli* BamC (dark blue) bound to BamD (2YHC [125]). The five TPR motifs of BamD coloured in light blue, yellow, green, pink and orange. (e) Lowest energy structure of *E. coli* BamE solved by NMR spectroscopy (2KXX [237]). All images were generated from the PDB files using the accession numbers given in brackets using UCSF Chimera molecular visualisation application [236].

Following chaperone-assisted transport across the periplasm, unfolded OMPs must insert and fold into the OM, a process which is assisted by the BAM complex [116]. The BAM complex in *E. coli* (Fig. 6a) is comprised of the outer membrane protein BamA (previously YaeT, Fig. 6b) and four accessory lipoproteins, BamB (YfgL, Fig. 6c), BamC (NlpB, Fig. 6d), BamD (YfiO, Fig. 6d) and BamE (SmpA, Fig. 6e) [105], [116]. BamA is an essential protein, the depletion of which causes an accumulation of aggregated OMPs in the periplasm, leading to cell death [117]. BamA belongs to the conserved Omp85 super-family first identified in *N. meningitides* [118]. Homologues are also found in mitochondria (Sam50) and chloroplasts (Toc75) [119]. All of the BamA homologues identified thus far have a C-terminal transmembrane β-barrel domain and a water-soluble N-terminal region [116]. The structure of the BamA β-barrel in two different organisms has been elucidated recently, revealing it to be 16-stranded with a conserved VRGY motif in loop L6 which is thought to be functionally important (Fig. 6b) [120].

The N-terminal soluble region of BamA comprises different numbers of polypeptide transport-associated (POTRA) domains, dependent on the identity of the organism. Bacterial BamA homologues tend to have five POTRA domains. Toc75 in chloroplasts, however, has three POTRA domains and Sam50 in mitochondria has only one POTRA domain [116]. Crystallisation of the four N-terminal POTRA domains (POTRAs 1–4) of *E. coli* revealed that despite their low sequence identity, the overall fold (a 3-stranded β-sheet with 2 α-helices) is conserved (Fig. 6b) [119]. Previous studies of the binding of OMPs revealed a direct interaction of BamA with unfolded OMPs and peptides mimicking the conserved aromatic-rich C-terminal sequence of OMPs [121]. Evidence of β-augmentation between POTRA domains of neighbouring proteins in the crystal structure of *E. coli* POTRA domains 1–4 suggests a possible mechanism for substrate binding [119]. Deletion of individual POTRA domains from BamA demonstrated that one of the functions of these soluble domains is to provide a scaffold for the association of the lipoproteins which make up the BAM complex with BamA [119]. Interestingly, while POTRAs 3–5 are essential for cell viability in *E. coli*, only POTRA 5 is essential in *N. meningitides* [116], suggesting differences in the roles of these conserved domains between bacterial species.

Of the four accessory lipoproteins in the BAM complex, only BamD has been shown to be essential for cell viability in *E. coli*, implying an important role for this lipoprotein in OMP biogenesis [116]. BamD is a predominantly α-helical protein with 5 tetratricopeptide repeat motifs (Fig. 6d), sharing structural similarity with other proteins which recognise C-terminal targeting sequences [122]. BamD interacts with BamA *via* POTRA domain 5 [116] and, accordingly, a mutation in POTRA 5 of BamA that is lethal at 37 °C causes dissociation of BamA from the BamCDE subcomplex [123]. Recent experiments *in vivo* using point mutations in BamD and BamA have suggested that BamD regulates the activity of BamA [123], [124]. Co-crystallisation of BamD with the non-essential lipoprotein BamC (Fig. 6d) revealed that a binding pocket in the N-terminal region of BamD is the site of interaction of these two lipoproteins [125]. A truncated variant of BamD was used to demonstrate binding of this lipoprotein to synthetic peptides based on the OMP C-terminal targeting sequence, and this interaction occurred at the same binding pocket as the interaction with BamC [116], [125]. Together, these data have led to the hypothesis that BamC may act as a regulator of BamD substrate binding [116], although the precise role of BamC (which has been shown to span the OM with parts of its sequence exposed to the extracellular environment (Fig. 6a) in OMP assembly remains unclear [126].

BamE (Fig. 6e) is the most recently discovered accessory lipoprotein of the BAM complex. This lipoprotein associates with the BAM complex *via* interaction with BamD and has been shown to cause only minor defects in OMP assembly when depleted [116], [127]. NMR spectroscopy revealed that BamE binds preferentially to PG lipids [127]. These lipids have been shown to enhance the insertion of OMPs into liposomes *in vitro* [116], suggesting that a role of BamE may be to promote OMP insertion into the OM. Interestingly, in *ΔbamE* and BamD R197L expressing strains of *E. coli*, BamA becomes more susceptible to degradation by proteinase K, suggesting that BamE might also be involved in conformational modulation of BamA [124], [128]. While BamC and BamE are associated with BamA *via* interaction of BamD with the soluble POTRA domains, BamB (Fig. 6c) [129], [130], [131], [132] is associated directly with BamA *via* POTRAs 2–5 (Fig. 6a) [116], [119]. Although not essential for cell viability, deletion of BamB results in reduced OMP assembly and a phenotype similar to SurA depletion mutants [133]. The OMPs most affected by deletion of BamB are those with larger β-barrels and, thus, it has been suggested that BamB has a role in substrate delivery to BamA, perhaps by increasing the substrate binding capacity of the BAM complex [116]. Indeed, BamB and BamD have been shown recently to bind to unfolded OmpA and unfolded BamA in the absence of the other BAM components [134]. Interestingly, both BamB and BamD accelerate BamA assembly into liposomes, however, OmpA folding was not similarly assisted by either lipoprotein, suggesting that one role of the BAM lipoproteins is to facilitate assembly of BamA [134].

Exciting recent work by Kahne and colleagues has demonstrated that the BAM complex can be reconstituted in a functional form *in vitro* [135]. The complex was found to have a 1:1:1:1 ratio of BamA:B:C:D, but the stoichiometry of BamE could not be determined due to its small size [135]. The activity of the BAM complex was monitored using the folding of the OM β-barrel peptidase enzyme, OmpT, which can be measured directly by cold SDS–PAGE or indirectly by following OmpT enzymatic activity [135], [136]. Compared with experiments in which the intact BAM complex was present, OmpT folding efficiency was reduced by approximately 6-fold in the absence of BamB [135], [136]. Reduced OmpT folding efficiency was also observed in the absence of SurA, even when the intact BAM complex was present [135], supporting the previous suggestion that BamB and SurA have similar, but not redundant, roles *in vivo* [116], [135]. These results also demonstrate that the BAM complex is able to complete the OMP assembly cycle without the aid of additional cellular components [136].

The mechanism of action of the BAM complex during OMP folding and membrane insertion is still under debate and several models have been proposed [116]. These include substrate translocation across the OM through BamA, followed by folding into the OM from outside the cell. Alternatively, the BamA β-barrel may act as a scaffold for β-sheet formation for the folding OMP or formation of BamA multimers may create a pore in the OM through which the substrate can insert [116]. Recent elucidation of the crystal structure of BamA from two different organisms, however, shows a large cavity in the BamA β-barrel within which a substrate could be accommodated [120]. Additionally, MD simulations point to the existence of a lateral gate in the BamA β-barrel, potentially allowing substrates to move from the barrel interior into the membrane [120]. The observation of two conformations of BamA with different resistance to proteinase K has been observed previously [124], [128]. These data and the observation that the conserved L6 loop is only accessible for labelling in one conformation [124], suggest that opening of the lateral gate may be modulated by movements of the L6 loop. Indeed, structural studies of BamA [120] and two homologues, FhaC [137] and TamA [138], revealed that the L6 loop adopts a different conformation in the FhaC crystal structure to that observed in the TamA and BamA crystal structures. This may suggest that L6 loop movements are a conserved functional feature across the Omp85 super-family. Lateral opening in TamA [138] has also been suggested MD simulations suggest, however, that FhaC does not share this functional feature [120]. These differences may be reflective of differences in substrate handling by these OMPs, with FhaC exporting substrates across the OM, while TamA and BamA most likely insert substrates directly into the OM. In contrast with the wealth of information about the mechanism of action of many cytosolic chaperones and chaperonins [41], [139], [140], there is clearly much to learn about the mechanism of action of the BAM complex and its associated periplasmic chaperones in facilitating the folding and membrane insertion of OMPs.

## Application of different biophysical methods to the study of OMP folding into lipid bilayers

In recent years, many techniques have been developed to study the folding of water-soluble proteins and together these have yielded a near-atomistic view of the folding landscapes of many such proteins [31]. By contrast, the experimental toolbox for the study of membrane protein folding is more limited [141]. Much of the information available about OMP folding into lipid bilayers has resulted from the use of techniques such as cold SDS–PAGE (where samples are not boiled prior to loading, resulting in the differential migration of the folded and unfolded conformations of the OMP [142]) and tryptophan fluorescence emission and far-UV circular dichroism (CD) spectroscopies on a limited subset of proteins, as summarised below and in Table 1. In spite of the challenges faced, the development of new methodologies and the application of existing methods to study OMP folding are now beginning to yield knowledge of OMP folding mechanisms both in the presence and absence of chaperones.

**Table 1:** Summary of selected *in vitro* folding studies of outer membrane proteins (OMPs) into lipid bilayers.

Protein	Notes	Techniques used	Selected References
OmpA	Folding studies of OmpA are described in this review and elsewhere.	Cold SDS–PAGE, far-UV CD, Trp fluorescence	Kleinschmidt (2006) [171]; Otzen (2013) [58]
PagP	Folding studies of a C-terminally his-tagged construct of PagP (HT PagP) and untagged construct (PagP) are described in detail in this review.	Cold SDS–PAGE, far-UV CD, Trp fluorescence, *Φ*-value analysis	Bishop et al. (2000) [239]; Ahn et al. (2004) [206]; Huysmans et al. (2010) [157]
hVDAC	Human voltage-dependent anion-selective channel (hVDAC) can be folded to the native state in LUVs composed of lipids of varying acyl chain length (*di*C_10:0_PC to *di*C_18:1_PC). Folding yield was estimated to be 94% in *di*C_12:0_PC LUVs. Secondary structure content of hVDAC in *di*C_12:0_PC LUVs was not affected by changing pH from 7.0 to 3.0.	Cold SDS–PAGE, far-UV CD, Trp fluorescence, sucrose density gradient centrifugation and proteolysis	Shanmugavadivu et al. (2007) [225]
FomA	FomA can be folded to the native state in both *di*C_10:0_PC and *di*C_18:1_PC (LUVs and SUVs). Kinetic analysis of folding into *di*C_10:0_PC and *di*C_18:1_PC SUVs suggested that FomA folds *via* parallel pathways into both lipids. The folding halftime is dependent on acyl chain length and reaction temperature.	Cold SDS–PAGE, Trp fluorescence, far-UV CD	Pocanschi et al. (2006) [202]
OmpG	The porin OmpG reconstituted in native *E. coli* lipids is gated by conformational changes in extracellular loops in a pH-dependent manner (closed at pH 5.0). Unfolding under force reveals each β-hairpin unfolds individually. Refolding from this mechanically unfolded state also proceeds by sequential folding of individual β-hairpins.	Atomic force microscopy (AFM)	Sapra et al. (2009) [240]; Damaghi et al. (2010) [241]; Mari et al. (2010) [242]; Damaghi et al. (2011) [243]
OmpF	Refolding of urea-solubilised OmpF into *di*C_14:0_PC SUVs occurred at only 15% yield. Refolding kinetics were biphasic but much slower than OmpA.	Cold SDS–PAGE, Trp fluorescence, far-UV CD	Surrey et al. (1996) [244]

As well as the methods mentioned above, the quenching of tryptophan fluorescence has been utilised successfully to follow the folding and membrane insertion of OmpA [143], [144]. In this technique, lipids which are brominated at different positions in the acyl chain are introduced into liposomes. The depth of membrane insertion of an OMP during a folding reaction is measured by following the kinetics of tryptophan fluorescence quenching by the bromine atoms within the bilayer [144]. Additionally, the use of mutagenesis to create OmpA variants with only a single tryptophan residue allowed the insertion of different regions of the OmpA β-barrel into the bilayer to be monitored. For example, varying the location of a single tryptophan residue can yield information on whether individual secondary structural elements are inserted sequentially or simultaneously [143]. Alongside other spectroscopic techniques, these experiments provided the first evidence of how an OMP folds, revealing that the folding and membrane insertion of OmpA is a concerted process [143], [144].

NMR studies on unfolded water-soluble proteins has provided evidence that the starting point of folding is not a random coil structure [22], [23], [28], [145], [146]. Indeed, residual structure in the unfolded state has been suggested to be important in initiating folding by facilitating the collapse of the polypeptide chain into conformations able to fold efficiently to the native state [22]. In contrast with helical IM proteins, OMPs can be denatured in urea or guanidine hydrochloride (GuHCl), enabling studies of their folding using classic Anfinsen-style experiments [43]. Analysis of the urea denatured state of OmpX has shown that the protein is globally unfolded, but has two regions of non-random structure: one a hydrophobic cluster and the other a helical region [147]. Analysis of peptides corresponding to these regions showed independent binding of the clusters to detergent micelles, suggesting a role of residual structure in the unfolded state in the initiation of OMP folding and membrane insertion [148]. The application of NMR spectroscopy to folded membrane proteins is complicated by the need to find a suitable mimic of the membrane environment. Detergent micelles are widely used, but problems with long term stability and the maintenance of proteins in a functional state are commonly encountered [149]. As a result, development of alternative non-micellar systems such as bicelles, nanodiscs and amphipols, to stabilise the folded state of membrane proteins has become an active research area [149], [150], [151] and is reviewed elsewhere in this issue.

Detailed information about the folding mechanisms of water-soluble proteins has been obtained by measuring the folding and unfolding kinetics of the protein of interest, using a spectroscopic probe to monitor the reaction time-course in the presence of varying concentrations of denaturant. The logarithm of the kinetic rate constants acquired from these data can be plotted against the denaturant concentration, giving a characteristic v-shaped chevron plot [152]. If the limbs of the chevron remain linear over denaturant concentrations from 0 M to the highest concentration used (highly denaturing) and the free energies associated with the folding and unfolding events equate to the equilibrium unfolding free energy, the data suggest that the protein under study probably folds *via* a two-state mechanism [13], [14]. Deviations from linearity, termed rollover, in either limb can occur and have been interpreted to indicate the presence of a folding intermediate [152], [153], movement of the transition state [154] or aggregation [155]. As well as being an indicator of folding mechanism (alongside equilibrium folding analysis), chevron plot analysis also yields information about the compactness of the transition state (or any populated intermediates) and their position on the folding reaction coordinate [152]. This detailed kinetic analysis is routine in the study of water-soluble protein folding and its recent application to study the folding of OmpA [156] and PagP [157] will be discussed later in this review.

**Fig. 7:**
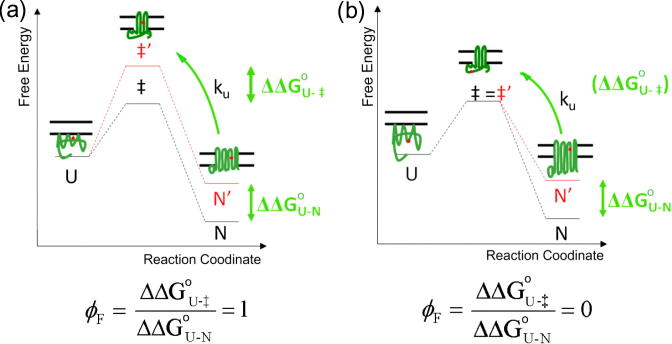
Schematic of the principles of *Φ*-value analysis. (a) A mutation (shown as a red dot) is made in a region of the protein which is native-like in the transition state (‡) leading to equal destabilisation of ‡ and the native state (N) resulting in a *Φ*-value of 1 or (b) a mutation is made in a region of the protein which is unfolded in ‡ but structured in the native state, leading to destabilisation of N only and a *Φ*-value of 0. It is assumed that the mutation does not affect the free energy of the unfolded ensemble (U). Abbreviations: ΔΔ*G*^°^_U–N_ refers to the difference in the free energy of folding upon mutation and ΔΔ*G*^°^_U–‡_ refers to the difference in the free energy between U and ‡ upon mutation. This image was adapted with permission from G.H.M. Huysmans.

Protein engineering methods coupled to kinetic analysis have been the most successful tool to probe the folding mechanisms of membrane proteins and have been applied to the IM proteins bR [44], [158], [159] (Fig. 2b), DsbB [160] and the OMP, PagP (Fig. 2d) [157]. *Φ*-Value analysis is a powerful protein engineering approach, which can be used to map the formation of contacts in the transition states and populated intermediates formed during protein folding (Fig. 7) [161], [162], [163]. In this approach, specific side-chain interactions are deleted by mutation, and the effect on the kinetic and thermodynamic parameters of the variant protein is measured and compared with wild-type [163]. The amino acid substitution may cause a change in the free energy of activation (kinetic), the equilibrium (thermodynamic) free energy of the folding reaction, or both. The ratio of these changes is the *Φ*-value, which is usually between 0 and 1, and gives a measure of the change of stability of the transition state (or intermediate), compared with the change in stability of the native state [163]. Both stabilities are measured relative to the denatured state, whose free energy is assumed to be unaffected by the mutation [163]. *Φ*-Values close to zero indicate that the transition state (or intermediate) is unstructured in the region of the amino acid substitution, while higher *Φ*-values indicate that the region is structured in the transition state (or intermediate) [161], [162]. Partial *Φ*-values are also observed and can be interpreted in several ways. For example, partial *Φ*-values may result if native contacts are partially formed in the transition state (or folding intermediate) or if multiple folding routes exist [163]. *Φ*-Values have provided a wealth of information on the structure of partially folded intermediates and transition states of water soluble proteins, revealing detailed information about their folding mechanisms [163]. Furthermore, the use of experimental *Φ*-values as constraints in MD simulations allows atomistic models of these ensembles to be created [164]. The main stumbling block for the application of *Φ*-value analysis to OMPs is the need to find experimental conditions that enable reversible folding for the protein of interest, which has proved difficult for many OMPs [58], [156], [165].

## Folding studies of OMPs *in vitro*

### OmpA

**Fig. 8:**
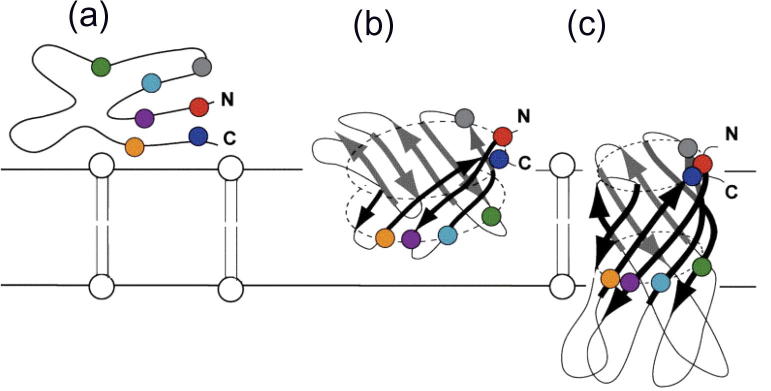
Proposed mechanism of folding and insertion of the OmpA β-barrel domain into lipid bilayers *in vitro*. (a) Depicts an unfolded, membrane-bound state, (b) depicts a partially folded and inserted state, and (c) depicts the native state. Coloured circles indicate the location of tryptophan residues in the OmpA structure. Reproduced from [238] with permission from Elsevier, © 2011.

The study of OMP folding *in vitro* has been pioneered by work on the monomeric, 8-stranded ion channel OmpA (Fig. 2e) from *E. coli*. Initial experiments using a combination of cold SDS–PAGE and protease digestion were conducted by Schweizer et al. as early as 1978 [166], which demonstrated that OmpA can fold in the presence of LPS and Triton X-100 detergent. It was not until over a decade later that Surrey and Jähnig reported the folding of OmpA into lipid bilayers [167], opening the door to studies of membrane protein folding in a more native-like environment. OmpA, solubilised in 8 M urea, was shown by these authors to insert spontaneously into small unilamellar vesicles (SUVs) of *di*C_14:0_PC upon rapid dilution from urea [167]. Detailed kinetic studies were carried out by these, and other, authors on the refolding of urea-unfolded OmpA into liposomes using cold SDS–PAGE, far-UV CD and tryptophan fluorescence emission [143], [168], [169]. Kinetic measurements of the formation of secondary structure by far-UV CD and tertiary structure by tryptophan fluorescence emission revealed that these structural elements form concomitantly [170]. Additionally, the rate of quenching of single tryptophan mutants of OmpA by brominated lipids suggested that the 8 β-strands penetrate the membrane simultaneously [144]. Based on these data a scheme for the refolding pathway of OmpA was proposed, beginning with collapse of the protein in aqueous solution, followed by adsorption to the membrane surface and folding to the native state by progressive penetration deeper into the membrane as the β-barrel forms (Fig. 8) [143], [144], [168], [169]. These experiments provided the first evidence of the concerted nature of OMP folding and membrane insertion [143], [144], [168], [169], [171].

More recently, measurement of the stability of OmpA has been achieved in several membrane mimetic environments [156], [172], [173]. Finding experimental conditions under which membrane protein folding is fully reversible and reaches equilibrium on experimentally tractable timescales *in vitro* is challenging and continues to impede studies of membrane protein folding and stability [174]. Folding of OmpA into SUVs composed of 92.5% C_16:0_C_18:1_PC and 7.5% C_16:0_C_18:1_PG was reported to be reversible [172]. Good agreement of the rate of folding when measured by tryptophan fluorescence emission and cold SDS–PAGE implied a two-state folding mechanism [172]. Introduction of guest lipids into this reversible system allowed the effects of bilayer thickness and the extent of lipid acyl chain saturation on OmpA stability to be measured, revealing that forces within the bilayer, such as curvature stress, affect thermodynamic stability [172]. Kinetic analysis of OmpA refolding into large unilamellar vesicles (LUVs) of varying diameter confirmed the role of curvature elastic stress in modulating the folding rate, suggesting that the irreversibility of folding observed for OmpA into LUVs may be a kinetic trapping effect [175].

Establishing conditions under which OmpA folds reversibly allowed double mutant cycle analyses of the role of side-chain-side-chain interactions in the protein folding mechanism [176], [177]. After investigating residues involved in OmpA function [176], this method was used to probe how the interactions between aromatic residues in the intrafacial region of the β-barrel contribute to stability [177]. In both α-helical and β-sheet membrane proteins, aromatic residues are enriched in the intrafacial region forming “aromatic girdles” [178]. This common feature of OMPs has been hypothesised to stabilise the β-barrel and to fix the location of proteins in the lipid bilayer, based on the partitioning energies of small model peptides into a model bilayer from aqueous solution [179]. Indeed, double mutant cycle analysis of OmpA provided the first evidence that clustering of aromatic residues provides a driving force for OMP folding and stability [177].

Analysis of OmpA folding into octyl maltoside micelles showed that, although OmpA folding remained irreversible when urea was used as a denaturant, reversibility could be obtained using GuHCl as an alternative denaturant [156]. A full kinetic characterisation of the folding mechanism was undertaken using chevron plot analysis and revealed that OmpA folds *via* a three-state mechanism to a native-like protein that has a thermodynamic stability much higher than that measured in SUVs (Δ*G*°_octylmaltoside_ ≈ −65.2 kJ/mol, Δ*G*°_SUVs_ ≈ −14.2 kJ/mol) [156], [172]. This result was confirmed in a subsequent study of OmpA folding into detergent micelles of *N*-lauryl-*N,N*-dimethylammonium-*N*-oxide (LDAO, Δ*G*°_LDAO_ ≈ −60 kJ/mol) [173]. By contrast, the thermodynamic stability of OmpA in the amphipol A8-35 was found to be lower than that in detergent SUVs (Δ*G*°_A8-35_ ≈ −8 kJ/mol), and the time taken to reach equilibrium during (un)folding in A8-35 was reduced to 25 days, compared with 52 days in LDAO [173]. These studies demonstrate the utility of different bilayer mimics to study membrane protein stability and function, allowing kinetic reversibility on an experimentally tractable timescale *in vitro*.

OmpA was also used in the first *in vitro* studies of the effect of a periplasmic chaperone on OMP folding into bilayers. Skp (Fig. 5b) was shown to maintain unfolded OmpA in a soluble state at low urea concentrations (≈0.4 M), to accelerate OmpA folding into negatively charged SUVs, but to retard folding into zwitterionic SUVs [180]. Additionally, pre-binding of the Skp:OmpA complex to LPS prior to addition of lipid was shown to promote folding into either negatively charged or zwitterionic SUVs suggesting that LPS may play a role in OMP assembly [180]. Incorporation of full length BamA into liposomes was found to increase the folding rate constant of the OmpA β-barrel domain into LUVs of *di*C_12:0_PC [181]. The transmembrane domain of BamA alone gave a small, twofold increase in the folding rate constant, which is comparable to the rate increase when FomA, an OMP not implicated in OMP assembly, is incorporated into the liposomes. This suggests that the rate increase observed with the BamA β-barrel alone results from non-specific perturbations of the local lipid environment [181]. This study also showed that incorporation of 20% *di*C_12:0_PE into the *di*C_12:0_PC LUVs has an inhibitory effect on OmpA folding which was overcome by the incorporation of BamA into the liposomes. These experiments suggest how OMPs may overcome the barrier to folding *in vivo* by destabilisation of the OM, which is composed of approximately 80% PE lipids, in the presence of BamA [181]. Finally, the previously observed retardation of OmpA folding into zwitterionic bilayers in the presence of Skp was shown to be ablated in the presence of BamA. This is possibly mediated through the interaction of negatively charged patches on the surface of the POTRA domains with positively charged Skp [181], [182]. Together, the available data on OmpA folding has provided valuable insights into the forces that govern the folding of OMPs. Lack of such extensive data on other OMPs, however, makes general conclusions hard to draw.

### Comparative studies of OMP folding

The study of homologous proteins has been utilised widely in the field of protein folding [183], [184]. Application of this approach to water soluble proteins includes the homeodomain-like super-family [16], [185], α-spectrin domains [37], [184], [186], [187], [188], [189], bacterial immunity proteins [190], [191], [192], ribosomal S6 proteins [193], [194] and immunoglobulin-like domains [195], [196], [197], [198], [199]. These studies have yielded valuable insights into the role of protein topology, amino acid sequence and secondary structure propensity on folding mechanisms [183], [184]. Recently, Burgess et al. adopted a screening approach using cold SDS–PAGE analysis to determine the folding yield of nine different OMPs (OmpX, OmpW, OmpA, PagP, OmpT, OmpLA, FadL, Omp85 (BamA) and OmpF) under different conditions [200]. The effects of pH, temperature, vesicle size and lipid composition were investigated, revealing that, in general, folding yield was increased by using pH values of 8–10, lipids with shorter acyl chains and smaller vesicle diameter [200]. These results accord with previous work on the effects of membrane thickness and curvature on the folding of OmpA [170]. A single condition to maximise the folding yield of all nine OMPs could not, however, be identified [200].

High temperature has been reported to increase the folding yield of OmpA [169], [201], FomA [202] and OmpX [201], but increasing temperature had varying effects on the folding yield of the OMPs in the Burgess study [200]. This variation was proposed to arise from the different aggregation propensities of the OMPs studied at higher temperature [200]. The aggregation propensity of the nine OMPs in the urea-unfolded state was investigating using sedimentation velocity analytical ultra-centrifugation, revealing that self-association did not correlate well with folding efficiency [203]. Some trends were observed, however, showing that addition of salt (50–400 mM) and lower pH (values 6–7) increase self-association, while the presence of ⩾4 M urea kept all the OMPs in a monomeric state [203]. The relatively low aggregation propensity of OmpA has since been attributed to the presence of the periplasmic domain, which has been shown to fold independently of the β-barrel domain [204]. A thorough screen to establish conditions for optimal folding of OmpLA was then conducted using the results of previous studies as a guide [200], [203], revealing that OmpLA folds reversibly into LUVs *di*C_12:0_PC at pH 3.8, 37 °C [165]. These conditions also allowed reversible folding of PagP and OmpW, but not the other OMPs studied [108]. The difficulty in finding conditions suitable for the folding of different OMPs is thus a challenge, making comparative studies of these homologous proteins challenging. It is therefore difficult to draw out similarities and differences in the folding mechanism of a single protein class in the context of OMP folding mechanisms.

### PagP

PagP is an OMP from *E. coli* that forms an 8-stranded β-barrel with a 19-residue amphipathic α-helix at its N-terminus (Fig. 2d) [205], [206], [207]. The β-barrel is tilted by approximately 25° to the membrane normal [205], [206], [208], stabilised in this position by the interactions of the aromatic girdles with the membrane intrafacial region [209]. PagP is a palmitoyl transferase enzyme, which transfers a palmitate chain from a phospholipid to hexa-acylated lipid A and thus helps to reinforce the structure of the outer membrane [210]. Folding studies on a C-terminally His-tagged construct of PagP (HT PagP) revealed that the protein folds into both detergent micelles and liposomes *in vitro* [211]. Unlike OmpA, a high concentration of urea (7 M) was required to solubilise the protein and prevent aggregation prior to insertion [143], [211]. Far-UV CD was used to follow the formation both β-sheet structure and tertiary structure (*via* a Cotton effect between tyrosine-26 and tryptophan-66) [211], [212]. In accordance with previous results on OmpA, secondary and tertiary structure were found to form concomitantly during HT PagP folding into either cyclofos-7 micelles or *di*C_12:0_PC liposomes (SUVs and LUVs) [170]. Mutants of HT PagP were then created to investigate the role of the N-terminal α-helix (residues 1–19) in folding, since the possession of a periplasmic α-helix is an unusual feature of an OMP [211]. This study demonstrated that the helix increases the stability of folded HT PagP in liposomes, but this effect was not so pronounced in detergent, illustrating the importance of the lipid bilayer in the stability of the native state and the importance of developing membrane-like mimics for studies of OMP stability [211]. One mutant in which a conserved residue in the α-helix, tryptophan-17, was replaced with alanine, unfolded fifty times more rapidly that the wild-type [211]. Moreover, in a HT PagP helix deletion construct, mutation of arginine-59, located in the intrafacial region of the β-barrel domain, to tryptophan restored the folding and unfolding kinetics in liposomes to rates similar to those of the wild type protein, demonstrating the importance of the aromatic girdle in the folding and stability of PagP [213].

**Fig. 9:**
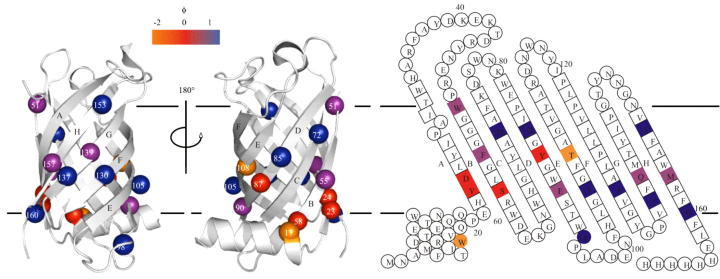
*Φ*-Value analysis of HT PagP. *Φ*_F_-values determined from kinetic analysis of HT PagP variants are mapped onto a ribbon diagram (left) and a topology model (right). Regions with *Φ*_F_-values close to 0 are shown in red, regions with *Φ*_F_-values close to 1 are shown in blue, intermediate *Φ*_F_-values are shown in purple, *Φ*_F_-values less than 1 are shown in orange and undetermined *Φ*_F_-values are grey. Reproduced with permission from [157].

By systematically varying the protein concentration and studying the folding of HT PagP under a range of lipid-to-protein ratios (LPRs), conditions were established under which the HT PagP unfolding transition is completely reversible in *di*C_12:0_PC LUVs [157]. Equilibrium stability studies and kinetic chevron plot analysis of HT PagP (un)folding revealed that the protein folds *via* a two-state mechanism over the range of urea concentrations studied (7.8–10 M). A *Φ*-value analysis was then undertaken for HT PagP using point mutants of 19 residues spread throughout the protein structure [157]. These experiments provided the first insights into the structural features of a transition state for OMP folding, suggesting a polarised transition state in which the N-terminal half of the protein remains largely unstructured, whilst the C-terminal half of the protein is native-like (Fig. 9) [157]. Interestingly, two negative *Φ*-values were observed, providing evidence for stabilisation of the transition state by non-native interactions [157]. The resulting mechanism of tilted insertion is consistent with the concerted folding and insertion suggested for OmpA [143], [144], [170]. It remains to be seen whether this mechanism is observed for other OMPs.

Kinetic analysis of HT PagP (un)folding over a range of urea concentrations (7.8–10 M) revealed that folding into *di*C_12:0_PC LUVs is best described by a burst phase followed by a single exponential phase [157], [211], [214]. Lowering the lipid-to-protein ratio, the HT PagP concentration, or inclusion of *di*C_12:0_PE in the *di*C_12:0_PC LUVs, resulted in a burst phase followed by two exponential phases [214]. Interrupted refolding experiments, in which folding is allowed to proceed for varying lengths of time before unfolding is re-initiated by dilution into high urea concentrations, were also carried out. These experiments revealed that the rate of formation of the native state is best described by two rate constants indicative of parallel folding pathways [214], a feature also seen in the folding of some water-soluble proteins [145], [197], [215], [216], [217], [218]. Since the unfolding kinetics of HT PagP are well described by single exponential kinetics under all conditions tested, there was insufficient evidence for an alternative native-like state of HT PagP [214]. The slower pathway most likely arises from a second population of folding-competent HT PagP molecules in solution ready to adsorb onto the lipid upon exposure of free membrane surface [214].

**Fig. 10:**
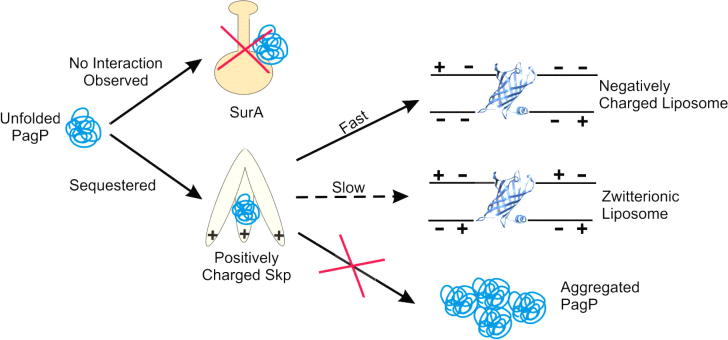
Schematic of the effects of Skp and SurA on the refolding of PagP. SurA and PagP do not interact stably under the conditions of the refolding assay. Skp readily interacts with PagP, retarding the PagP folding rate into zwitterionic liposomes, but accelerating the folding rate of PagP into negatively charged liposomes in a manner dependent on the ionic strength of the buffer. Additionally, the holdase activity of Skp was demonstrated by its ability to rescue the folding and membrane insertion of HT PagP under conditions which strongly favour aggregation of this construct. This figure was adapted from [92] with permission from Elsevier, © 2013.

More recently, kinetic analysis of an untagged variant of PagP has been undertaken in the presence of SurA and Skp [92]. This PagP construct, initially reported by Burgess et al. [200], was chosen for folding assays in the presence of soluble chaperones as it had been reported to be folding competent in urea concentrations as low as 1 M, in stark contrast with the high urea concentrations required for efficient folding of HT PagP [200], [211]. Interestingly, however, folding of PagP is not fully reversible under conditions which promote reversible folding of HT PagP, again highlighting the difficulty in generating OMPs suitable for equilibrium denaturation studies [92]. Nonetheless, the folding kinetics of PagP into both zwitterionic and negatively charged liposomes were investigated in the presence and absence of Skp or SurA (Fig. 10) [92]. These experiments showed that membrane composition and ionic strength of the buffer strongly influences the effect that Skp has on PagP folding, suggesting that electrostatic interactions play an important role in the mechanism of action of this chaperone [92], consistent with the previous results on Skp-mediated folding of OmpA [92], [182]. SurA, however, did not affect the observed folding rates of PagP, in contrast with the results observed for OmpT refolding [135], but consistent with the view that Skp and SurA may act by distinct mechanisms in partially redundant chaperone pathways [78], [105]. The ability of Skp to prevent the aggregation of HT PagP was also investigated, revealing that even under conditions in which aggregation is strongly favoured, Skp can rescue the folding and membrane insertion of HT PagP [92]. Together, these studies indicate the power of combining different methods to study the folding mechanism of an OMP and set the scope for future investigations into how OMPs fold both unassisted and assisted by folding factors *in vitro* and *in vivo*.

## Summary and outlook

Early experiments on the folding of water-soluble proteins focused on small, single domain proteins as these provide relatively simple folding models [3], [11], [13], [14]. In the intervening decades, many details on the folding of these proteins have been elucidated as new experimental methods and computational techniques have been developed [31]. More complicated folding scenarios are beginning to be investigated experimentally, including studies of multidomain proteins, oligomeric proteins, folding in the presence of chaperones and in the cellular milieu [40], [41], [139], [219]. Even fifty years after Anfinsen’s experiments, the first simulations of protein folding in all-atom detail have only been reported in the last two years and these initial studies were limited to small (<100 residues), single domain proteins [30], [220], [221]. More recently, the characterisation of the folding and assembly of a dimeric protein in atomistic detail using equilibrium MD simulations has paved the way for simulation studies of the folding of oligomeric proteins [222]. Application of simulation techniques to study integral membrane proteins firstly requires the protein under study to be correctly positioned within the bilayer as this information is not obtained when membrane protein structures are determined by X-ray crystallography or NMR [223]. This is often achieved by first applying a coarse-grained approach to correctly position the protein within the membrane and using these results to guide atomistic simulations to obtain detailed information on lipid-protein interactions and protein dynamics [223]. The multiscale simulation approach has been successful in probing the lipid interactions of aquaporins, ion channels and G-protein coupled receptors, among others, but simulating the complexities of lipid organisation within membranes remains a challenge in this field [223], [224].

Similar to the evolution of studies of water-soluble protein folding, analysis of OMP folding mechanisms, thus far, have focused on a small subset of relatively simple proteins that are amenable to the array of kinetic and thermodynamic assays required to determine folding mechanisms [58], [159]. Recent trends, however, suggest that the field of membrane protein folding is already progressing towards more complex folding systems. Folding models utilised thus far include OmpA and Omp85 (a BamA homologue) both of which have soluble, periplasmic domains, the trimeric porin, OmpF and the large, 19-stranded hVDAC [108], [200], [203], [225]. Moreover, the use of periplasmic chaperones to aid folding and membrane insertion has now been applied to several different folding systems [92], [108], [135], [182].

Membrane protein folding is complicated by the need to recreate a suitable membrane mimetic environment *in vitro* into which the protein can fold [47]. While early studies concentrated on simple micellar systems, these often do not provide a good mimic for the complex, heterogeneous environment of a membrane *in vivo* and can lead to inactivation or aggregation of the protein of interest [226]. The use of lipid-based mimics, usually synthetic liposomes, has provided a wealth of information based on the ability to introduce guest lipids to modulate surface charge, lateral pressure and membrane fluidity and to examine their effect on OMP folding and stability. Studies to date have focused on relatively simple lipid mixtures, often with short acyl chains (∼C_12_), as these have been shown to promote the folding of OMPs *in vitro*, and at lipid-to-protein molar ratios (often 400:1 to 3200:1, or up to approximately 100:1 by weight) which far exceed those encountered in biological membranes (in the range 1:1 to 1:3 by weight [227]). While much has been learned about the interactions between OMPs and their surrounding lipid environment from these studies, these simple mimics do not accurately represent the folding situation *in vivo*. Indeed, the membrane environment of OMPs *in vivo* is characterised by variable lipid composition and asymmetric distributions of lipids, as well as being densely packed with the many proteins associated with, and inserted into, the bilayer [63].

As the experimental toolbox for the folding of OMPs continues to expand, the level of complexity attainable in *in vitro* models is likely to increase. Additionally, as the gap in understanding between the folding of OMPs and water-soluble proteins continues to decrease, the similarities and differences between the folding mechanisms of these two protein classes will emerge. This knowledge will be critical in understanding some of the fundamental biophysical questions which remain to be answered, such as the relationship between the primary sequence of a protein and its native structure in the context of water or lipid as the solvent, and how chaperones assist folding *in vitro* and *in vivo*. Moreover, these concepts will be critical in refining current knowledge of protein folding mechanisms and working towards a universal folding theory which encompasses all of nature’s proteins.

## Footnotes

1 *Abbreviations used:* bR, bacteriorhodopsin; PDB, protein data bank; hVDAC, human mitochondrial voltage-dependent anion-selective channel; NMR, nuclear magnetic resonance; IM, inner membrane; OM, outer membrane; PE, phosphatidylethanolamine; PG, phosphatidylglycerol; LPS, lipopolysaccharide; OMP, outer membrane protein; PPIase, parvulin-like peptidyl-prolyl isomerase; BAM, β-barrel assembly machinery; MD, molecular dynamics; POTRA, polypeptide transport-associated; CD, circular dichroism; SUV, small unilamellar vesicle; LUV, large unilamellar vesicle; C*_x_*_:_*_y_*PZ, a phospholipid where *x* indicates the number of carbon atoms in the acyl chain, *y* indicates the number of double bonds per acyl chain and Z represents the head-group moiety; PC, phosphatidylcholine; LDAO, *N*-lauryl-*N,N*-dimethylammonium-*N*-oxide; HT PagP, C-terminally His-tagged PagP; SDS–PAGE, sodium dodecyl sulphate polyacrylamide gel electrophoresis; GuHCl, guanidine hydrochloride.
